# New Insights on Plant Cell Elongation: A Role for Acetylcholine

**DOI:** 10.3390/ijms15034565

**Published:** 2014-03-17

**Authors:** Gian-Pietro Di Sansebastiano, Silvia Fornaciari, Fabrizio Barozzi, Gabriella Piro, Laura Arru

**Affiliations:** 1 DiSTeBA (Dipartimento di Scienze e Tecnologie Biologiche ed Ambientali), University of Salento, Campus ECOTEKNE, Lecce 73100, Italy; E-Mails: gp.disansebastiano@unisalento.it (G.-P.D.S.); gabriella.piro@unisalento.it (G.P.); 2 Interdepartmental Research Centre Biogest-Siteia, University of Modena and Reggio Emilia, Reggio Emilia 42122, Italy; E-Mail: silvia.fornaciari@unimore.it; 3 Department of Life Science, University of Modena and Reggio Emilia, Reggio Emilia 42122, Italy; E-Mail: barozzi.fabrizio@virgilio.it

**Keywords:** cell elongation, acetylcholine, expansin, protoplasts, endocytosis, vesicle trafficking, vacuoles

## Abstract

We investigated the effect of auxin and acetylcholine on the expression of the tomato expansin gene *LeEXPA2*, a specific expansin gene expressed in elongating tomato hypocotyl segments. Since auxin interferes with clathrin-mediated endocytosis, in order to regulate cellular and developmental responses we produced protoplasts from tomato elongating hypocotyls and followed the endocytotic marker, FM4-64, internalization in response to treatments. Tomato protoplasts were observed during auxin and acetylcholine treatments after transient expression of chimerical markers of volume-control related compartments such as vacuoles. Here we describe the contribution of auxin and acetylcholine to *LeEXPA2* expression regulation and we support the hypothesis that a possible subcellular target of acetylcholine signal is the vesicular transport, shedding some light on the characterization of this small molecule as local mediator in the plant physiological response.

## 1. Introduction

Cell elongation is a process known to be dependent on auxin. Although it has been studied for decades the mechanism is still not completely unravelled, since the response of the cell to the elongation stimulus is integrated and highly complex [1,2]. Recently, it has been proposed that auxin transport could involve secretion via an endocytic vesicle recycling process, at least at the root apex [3–8]. This vesicular secretion closely resembles the synaptic communication in animals [9–12] and, at the same time, supports the neurotransmitter-like concept of auxin [13]. Plants possess many homologous molecules that are similar to the neurotransmitters of the animal nervous system such as glutamate, GABA, serotonin, melatonin, dopamine, and acetylcholine [14–19]. The role of these molecules in plants is still far from understood [20–23]. These compounds can act as deterrents against predation, but they may somehow provide a signalling function also in plants. Supporting this role, receptors have been found [24,25]; among them the glutamate receptors are the better characterised (for review [26]). Serotonin and melatonin [21–23], as well as l-glutamate [27], appear to regulate the root system architecture. Acetylcholine (ACh) seems to mediate various physiological processes, such as phytochrome-based signalling [28–30], water balance [31], cell swelling [32,33], stomatal movement [34,35], root-shoot signal transduction [36] and cell elongation [37,38].

Expansins are well known key enzymes involved in cell elongation. They are encoded by a multigene family with unique and well-defined cell wall loosening effects, mediating cell wall relaxation in a pH-dependent manner [39] and enabling plant cells to elongate in response to their internal turgor pressure. *LeEXPA2* belongs to the expansins multigene family, and it is up regulated by auxin in the young growing regions of tomato hypocotyl [40,41]. We previously investigated the role of auxin and sugars on *LeEXPA2* mRNA expression in elongating tomato hypocotyl segments [42]. Sugar cross-talk with hormonal signalling is well documented [43–45]. Sugars are carbon and energy source in cell metabolism, but they are also a signal informing the plant about its metabolic state, affecting physiological processes such as germination, flowering and senescence [43,44,46–49].

In the present work we used *LeEXPA2* expression to investigate the effect of auxin and ACh on cell elongation. Our research focuses on cell elongation with two main goals: to discriminate between the contribution of auxin and ACh on *LeEXPA2* gene expression, and to identify the subcellular target of ACh signal in vesicular transport, contributing to the characterization of this kind of small molecule as local mediator in the plant physiological response.

## 2. Results

### 2.1. Determination of ACh Effect on Hypocotyl Segment Elongation

**Figure 1.**
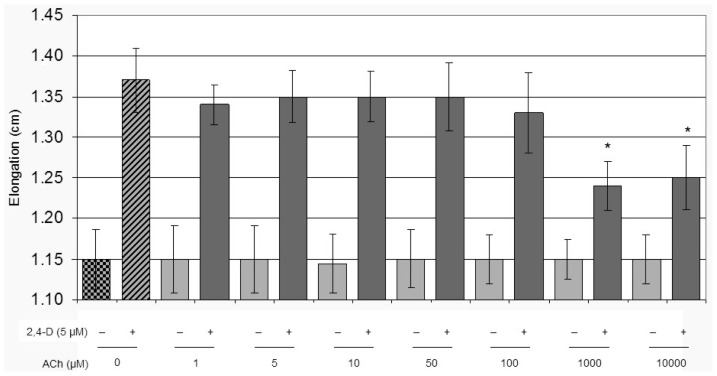
Effects of acetylcholine on tomato hypocotyl segments elongation. Hypocotyl segments were incubated for 16 h in buffer and increasing concentrations of acetylcholine (ACh) as indicated in the figure, with or without 5 μM 2,4-D. Hypocotyl segment length data are mean ± SE, *n =* 20. Group means were analysed by ANOVA followed by Tukey test (* *p <* 0.05).

We investigated the effect of different concentrations of ACh alone or combined with a constant amount of the synthetic auxin 2,4-D (2,4-dichlorophenoxyacetic acid) on hypocotyl segment elongation (Figure 1). The ACh dose definition was determined incubating tomato hypocotyl segments for 16 h. ACh concentrations higher than 100 μM spoiled the positive effect of 2,4-D, inhibiting segments elongation in presence of auxin. Considering that we do not know how ACh could be taken up from the medium by the plant tissue, a concentration of 50 μM ACh was chosen for further experiments. From our previous work [42] we already know that if the segment elongation has to be monitored, the presence of sucrose is an essential requirement to sustain the auxin-induced *LeEXPA2* transcript in a 16 h-long experiment. We are also aware that sucrose is more than an energy supply in the auxin induced expansin transcription [41,42], and we decided to take these observations into account in all the experimental conditions.

### 2.2. ACh Alone Has a Moderate Induction Effect on LeEXPA2 Expression, but a Very Strong Effect When Combined with Auxin

**Figure 2.**
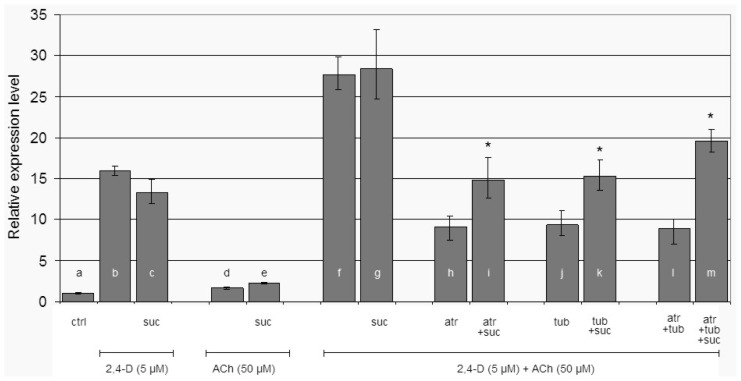
Relative *LeEXPA2* gene expression level. Relative *LeEXPA2* gene expression in tomato hypocotyl segments after 2 h of incubation at different conditions as indicated in the figure. Ctrl = control in buffer alone; suc = sucrose (60 mM); atr = atropine (0.1 μM); tub = tubocurarine (0.1 μM). Pair of treatments (with or without sucrose) were analysed by ANOVA followed by Tukey test (* *p <* 0.05).

In order to avoid the effect of starvation we quantified *LeEXPA2* transcript level in hypocotyl segments after 2 h of incubation. The possible signalling contribution of sucrose was taken into account quantifying *LeEXPA2* in response to 2,4-D, ACh and their combination, with or without sucrose (Figure 2). There was no statistically significant difference in *LeEXPA2* mRNA accumulation between segments treated with 2,4-D or 2,4-D plus sucrose (Figure 2, histograms b,c). The supplying of ACh without or with sucrose does not affect *LeEXPA2* transcription (Figure 2, histograms d,e), but when ACh was supplied together with 2,4-D, or with 2,4-D plus sucrose, the effect was more than simply additive (Figure 2, histograms f,g).

The specificity of ACh on gene induction was investigated by supplying two known antagonists of animal ACh receptors: atropine (atr) [50] and tubocurarine (tub) [51]. The observed effect was to decrease *LeEXPA2* transcript to about 33% of the mRNA level registered in the relative control without antagonists (Figure 2, histograms h,j,l *vs.* f). *LeEXPA2* mRNA was assessed to about 52% in presence of the single inhibitors when sucrose was added (Figure 2, histograms i,k *vs.* g), and to about 69% when the inhibitors were supplied together (Figure 2, histogram m *vs.* g). In all these last three different conditions (single inhibitors or the combination of both), sucrose statistically made the difference in maintaining expansin transcript.

### 2.3. Auxin and ACh Interfere with Endocytosis but Promote Early Steps of the Process

Hypocotyl elongation is certainly related to endomembrane traffic; synergistic effects of auxin and ACh may be explained by an effect on such traffic. We prepared protoplasts from etiolated hypocotyls isolated from seedlings grown in the same physiological conditions maintained in the other experiments and we loaded these cells with the endocytic tracer FM4-64. Protoplasts are cells deprived of their rigid cell wall that strongly influence plasma membrane turnover.

**Figure 3.**
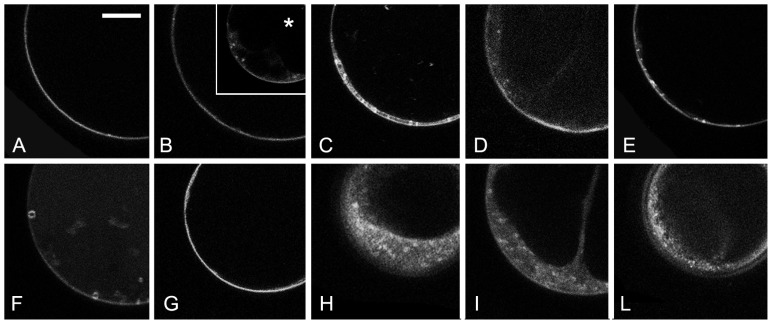
FM4-64 internalization in tomato hypocotyl protoplasts. (**A**–**E**) Images of protoplasts within the first 20 min of staining. (**A**) In control conditions labelling remained essentially limited to the PM in all large cells; (**B**) Effect of BFA treatment appeared to reduce FM4-64 internalization in large cells even if in small cells BFA bodies rapidly appeared (inset *); (**C**) Effect of treatment of protoplasts with 5 μM Auxin; (**D**) Effect of treatment with 50 μM ACh; and (**E**) Effect of combined chemicals; (**F**–**L**) Images of protoplast after 80 min of staining. (**F**) Control protoplast with defined late endosomes; (**G**) BFA treated cell with no internalization of the dye; (**H**) Protoplast treated with Auxin; (**I**) Protoplast treated with ACh; and (**L**) Protoplast treated with combined chemicals. Scale bar: 10 μm.

The prepared protoplasts had no visible plastids or rare etioplasts with a visible epifluorescent prolamellar body (not shown). Two sub-populations of cells, with large (above 30 μm) and small diameter (below 30 μm) could be distinguished. The few minutes time, required for the slide preparation, was sufficient to allow the loading of cell plasma membrane (PM) with FM4-64 and occasionally very small endocytic compartments were also visible within 10 min. Nonetheless, within the first 20 min, the labelling remained essentially limited to the PM in all large cells (Figure 3A). Small cells appeared to have a more active endocytosis with an earlier appearance of internal dotted structures (not shown). Brefeldin-A (BFA) treatment made more evident FM4-64 internalization in small cells, probably blocking the dye in newly forming BFA bodies (Figure 3B inset) but appeared to reduce the dye internalization in large cells (Figure 3B). It is known that BFA may have very specific effects in different cell types and also inhibit endocytosis [52,53]. We therefore decided to limit our observation to cells with a diameter above the 30 μm limit where endocytosis was inhibited and differences were more evident.

The chemical protoplasts treatment with 5 μM 2,4-D (Figure 3C), 50 μM ACh (Figure 3D) or the combination of both (Figure 3E) induced an increase of very small dotted structures visible immediately after FM4-64 loading. After 80 min of FM4-64 internalization, control protoplasts presented well defined late endosomes with a diameter over 1 μm (Figure 3F), while BFA treated cells showed almost no internalization of the dye (Figure 3G). The treatment with the synthetic auxin (Figure 3H), ACh (Figure 3I) or the two combined chemicals (Figure 3L) inhibited the correct endocytosis inducing an aberrant FM4-64 distribution pattern that appeared almost diffused in the cytosol.

### 2.4. Auxin and ACh Affect the Compartment Involved in Golgi-Independent ER-to-Vacuole Transport

**Figure 4.**
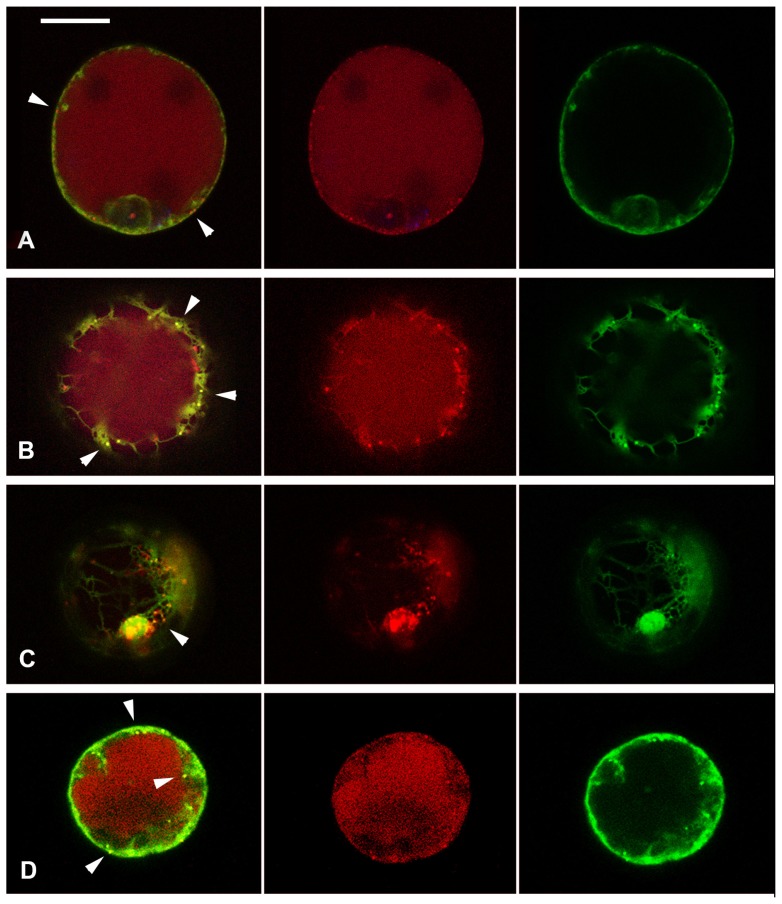

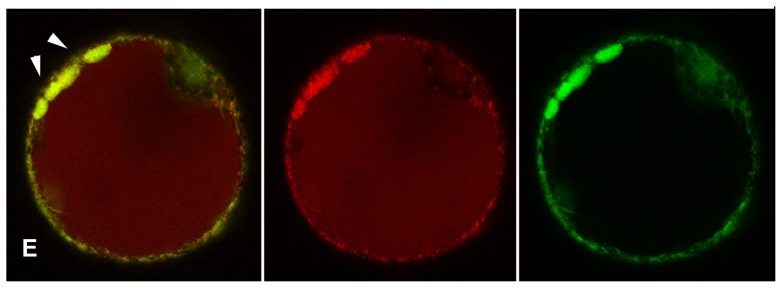
Tomato hypocotyl protoplasts transiently co-transformed with GFPChi (in green colour) and AleuRFP (in red colour). (**A**) Cell in control conditions, GFPChi is restricted to the ER and only AleuRFP labels small punctate structures (white arrows); (**B**) Cell treated with Auxin, it induces concentration of GFPChi in small punctate structures that colocalized partially with those labeled by AleuRFP (white arrows); (**C**) Cell treated with ACh, it caused accumulation of GFPChi in small vacuoles surrounded by several AleuRFP labeled structures (white arrow); (**D**) Cell treated with the two chemical combined, the treatment appeared very stressful but again it was evident that GFPChi and AleuRFP colocalized in punctate structures (white arrows); and (**E**) Cell treated with BFA induced bodies where GFPChi and AleuRFP colocalized partially (white arrows) but colocalization was not observed in the small discrete compartments exclusively labeled by AleuRFP. Scale bar: 20 μm.

The central vacuole is essential to drive plant cell expansion [54]. For this reason, the two well-documented vacuolar fluorescent reporters AleuRFP and GFPChi [55] were expressed in tomato hypocotyl protoplasts to visualize vacuoles and to monitor auxin and ACh effects on endocytic compartments organization. GFPChi is the result of the fusion of GFP (Green Fluorescent Protein) with the *C*-terminal VSD of tobacco chitinase, while AleuRFP comes from the *N*-terminal fusion of the Red Fluorescent Protein (RFP) with the sequence-specific VSD of the barley cysteine protease aleurain. It has been reported that AleuRFP transits through a prevacuolar compartment (PVC), which is identified as late endosome (LE), while GFPChi reaches the vacuole bypassing Golgi stacks [56]. In *Arabidopsis* [55] and *Tobacco* [56] protoplasts, GFPChi and AleuRFP partially colocalize in the central vacuole (Lytic Vacuole = LV). In tomato hypocotyl protoplasts AleuRFP was distributed as expected in PVC and LV while GFPChi was very rarely observed in the LV and its localization was generally restricted to the Endoplasmic Reticulum (ER) (Figure 4A).

All chemical treatments visibly stressed the cells. When 2,4-D was applied, GFPChi distribution was altered and the reporter was concentrated in small punctate structures that colocalized partially with AleuRFP positive PVC (Figure 4B). ACh caused accumulation of GFPChi in small compartments, large enough to be named vacuoles, surrounded by many PVC that appeared to be associated (Figure 4C). The two chemicals combined were very stressful for the cells, but it was evident that again GFPChi and AleuRFP colocalized in PVCs (Figure 4D). BFA treatments induced BFA-bodies where GFPChi and AleuRFP partially colocalized similarly to that observed in tobacco [56]. Colocalization was not observed in the small discrete compartments that may be identified as PVCs (Figure 4E) where, on the contrary, the two fluorescent markers colocalized during auxin and ACh treatments (Figure 4B–D).

## 3. Discussion

Expansins were first identified by McQueen-Mason and colleagues [57] and are members of a multigene family known to be cell-wall-loosening enzymes. They allow turgor-driven cell expansion by disrupting hydrogen bonds of the wall polysaccharides, and they are involved in a variety of physiological responses such as root growth [58], internode elongation [59], leaf development [60], fruit development [61], floral development [62], fruit maturation [63,64], and seed germination [65]. They are expressed in the apical meristem [66] and in active elongating tomato hypocotyl tissues [41,42,67]; they also play important roles during abiotic stress response [68–70]. Expansin activity is shown to be regulated by many factors. The promotor region includes target sites for transcription factors regulated by hormones such as auxin [67,71], gibberellins [72,73], cytokinin [74,75], ethylene [76,77], or by light [41].

In the experimental system based on tomato elongating hypocotyl segments used here, *LeEXPA2* is induced by auxin and it is directly involved in cell elongation confirming what was previously reported [40–42,67]. After 2 h of treatment, there is no statistically significant difference in *LeEXPA2* transcript amount between hypocotyl segments subject to 2,4-D, or 2,4-D plus sugar (Figure 2). This observation may indicate that sugar has no signalling role on its own, or–at least–that it is fully masked by auxin effect. When ACh is added to the medium, the presence of sucrose seems to cause a slight increase in *LeEXPA2* mRNA amount. Sucrose has proven to possess a signalling role in many cases [78–81], but for a molecule so tightly integrated in the plant cell metabolism it is difficult to separate its contribution as metabolic/energetic compound from the signalling one. Considering *LeEXPA2* transcript level accumulation upon ACh or auxin stimulation (Figure 2), while ACh alone does not seem to induce any changes in *LeEXPA2* transcript level, the combination of both auxin and ACh causes a strong increase in expansin transcript abundance, more than additive when compared to the effect due to the single chemicals.

ACh has already been shown to possess some of the effects historically ascribed to auxin, such as rooting of leaf explants [82], promoting lateral roots [83], interacting with phytochrome [28], and mediating coleoptyl segment elongation [37,38]. In our experimental system, no additive effect was observed in segment elongation when auxin and Ach—with or without sucrose—were added to the medium (data not shown); maximum elongation after 16 h was observed in hypocotyl segments treated with auxin alone. Our observation differs from Lawson and colleagues [38], who reported that higher concentration of ACh had an inhibitory effect on elongation. In any case, the literature does not provide support in the definition of an optimal physiological concentration of ACh in plants. A wide range of ACh concentration is described, from 10 μM in oat coleoptile segments [37], to 10^−4^–10^−2^ M in pea chloroplasts [84], 10^−8^–10^−2^ M in wheat protoplasts [85], 23 pmol/g in *Arabidopsis* [86], and 2.94 μmol/g in *Bamboo* shoot [86,87]. An even wider range of ACh concentration was used in various experimental systems: 0.3 mM on germinating beans [29], 0.1 mM on oat coleoptile segments [37], 10 μM on peels from *Vicia faba* leaves [35], and 1 nM on germinating radish [83]. We failed to measure the endogenous concentration of ACh in tomato hypocotyls; therefore we monitored the effect of ACh on hypocotyl segments elongation in order to identify an optimal concentration for the experiments (Figure 1). An ACh concentration beyond 50 mM appeared to negatively interfere with the auxin driven elongation process, supporting the observations of Lawson and colleagues (1978) [38]; we therefore adopted the concentration just below (50 mM). Further experiments are needed to understand whether smaller concentrations may be sufficient to induce expansin transcription.

The ACh effect described here is specific. The elongation induced by the combined action of ACh and auxin, either in presence or in absence of sucrose, was prevented by adding to the medium the inhibitors of mammal ACh receptors (Figure 2). The evidence of a cholinergic system in plants is still lacking [14], but ACh and the enzymes necessary to its biosynthesis and degradation are already been found in life forms without a mammal-like nervous system, such as bacteria, fungi, yeast, and finally plants [31,86]. The application of specific agonists such as muscarin and nicotine, and antagonists such as atropine and tubocurarine [50,51] indirectly suggests the possible existence of the two main receptors types (muscarinic and nicotinic). Since plants possess all the neurotransmitter-like molecules proper of the mammalian nervous system, such as dopamine, melatonin, serotonin, GABA, and glutamate [14,15,17,18,88,89], it is reasonable to hypothesize that these compounds could be involved in some kind of signal transmission [18,90,91] in addition to a possible metabolic role. In some cases these molecules act as deterrents for plant predation, but for some of them the presence of plant-specific receptors has been proven [24–26,92]. Atropine and tubocurarine efficacy in our experimental system might support the indirect evidence of the existence of an ACh receptor: a receptor that may be competitively bound by selective antagonistic compounds.

We also investigated the modification of the cell upon auxin and ACh stimulation, observing the alteration of membrane and vesicular traffic. It has already been proven that cell-to-cell auxin transport depends on a vesicular traffic, and that auxin loading involves carriers derived from plasma membrane and endosome recycling [18,19,93]. In human and animal systems, ACh is also packaged into vesicles after been synthesized. ACh vesicular import occurs by means of a carrier protein on the vesicle membrane. Once packaged, ACh is stored into the synaptic vesicles pool [94–96]. Synergistic effects of auxin and ACh may be explained by an effect on membrane traffic; we used protoplasts from etiolated hypocotyls to test this hypothesis. Protoplasts are expected to provide a reasonable indication of plasma membrane turnover and endomembrane organization in the differentiated cells of the studied tissue [97–99].

Protoplasts from etiolated hypocotyls, similarly to other protoplasts populations [55,100], had no less than two distinct sub-populations: one with large diameter (above 30 μm), the other with small diameter (below 30 μm). Being aware that vesicle traffic and endomembrane organization may be different, and that BFA (chosen as control treatment) may have very specific effect in different cell types [53], we decided to limit our observations to cells with a diameter above the 30 μm. Protoplasts were stained with the styryl dye FM4-64, commonly used as endocytic tracer [101–103] and a BFA treatment was used as a control of the cell behaviour. We found that the drug had different effects on the two size-based subpopulations of the cells derived from tomato hypocotyl. While BFA induced rapid accumulation of the dye in small cells, it inhibited FM4-64 internalization in large cells. Several authors stressed that BFA action is determined by the localization and concentration of resistant and sensitive ARF (ADP-ribosylation factor)/ARF-GEF (Guanine nucleotide Exchange Factor) complexes and can have different effects in different tissues [52]. On the contrary, the treatments of cells with 2,4-D 5 μM, ACh 50 μM or the two combined chemicals, induced also in large cells a rapid increase of very small dotted structures immediately visible after FM4-64 loading. Staining of the PM drastically decreased indicating a clear acceleration of the dye internalization. The observed increased internalization was anyhow different from the normal endocytotic process. In fact, after longer internalization time, when well-defined late endosomes should have been visible, the correct endocytosis progress appeared to be impaired. 80 min after FM4-64 loading, the marker did not progress in the internal membranes, especially the late endosome; on the contrary it was trapped in very small vesicles dispersed in the cytosol. The normal progression of the endocytotic pathway was altered. Both auxin and ACh did not prevent FM4-64 internalization but affected a second step of endocytosis leading to large late endosomes. BFA treated cells provided a control situation in which almost no internalization of the dye was observed. In this case the dye remained confined to the PM. This control was useful to clearly distinguish auxin and ACh effect from a simple endocytosis inhibition. The existence of several endocytotic and recycling pathways was proven by the production of functional BFA-sensitive or BFA-resistant ARF-GEFs variants [104]. It was shown that BFA-sensitive GNOM regulated PIN1 and PIN3 recycling [104,105], but it was not involved in the recycling of AUXIN-RESISTANT1 (AUX1), PIN2, and PM-located H^+^-ATPase [106] to the PM. The possibility of differential effects suggests the existence of multiple functionally distinct early/recycling endosomes (REs). GNOM partially co-localizes with FM4-64 and REs might then be part of the Trans Golgi Network (TGN) as suggested by [104], nonetheless the two compartments have a different sensitivity to BFA [106].

Certainly the auxin and ACh effect is different from the effect of BFA. BFA disrupts Golgi-based secretion and leads to the formation of endomembrane bodies (BFA bodies) that incorporate early endosomes and TGN components as well as Golgi resident proteins. BFA also aggregates the endosomal population of BRI1 [107,108] and disrupts the endocytosis of PIN2, AUX1, PIN1 and PIN7 [105,106,108]. We speculate that the very small FM4-64-labelled dots visualized during auxin and ACh treatments are compartments characterized by the transit of PIN1 and PIN7 [106,107].

Looking for further information on the effect of auxin and ACh treatments on late endosomal compartments, we investigated post-Golgi compartments, labelling them with two known vacuolar markers: GFPChi and AleuRFP [55]. These reporter proteins follow two different and independent routes to the vacuole [55,56,100]. BFA also had a differential effect on the two markers. The inhibition of COPII trafficking by a specific dominant-negative mutant (NtSar1h74l) confirmed that GFPChi transport from the ER to the vacuole is not fully dependent on the Golgi apparatus [56]; suggesting the existence of a not yet characterized intermediate compartment. On the contrary, AleuRFP/GFP transits through Golgi stacks. In *Arabidopsis* [55] and *Tobacco* [56] protoplasts, GFPChi and AleuRFP partially colocalize in the central vacuole (Lytic Vacuole = LV) but never colocalize in the intermediate compartments [56]. In tomato protoplasts from etiolated hypocotyls GFPChi was never observed in the LV. In the absence of a biochemical evaluation, this is not the definitive indication that this marker does not reach the LV because proteases and low pH may reduce GFPChi fluorescence in the vacuole below detection limits [109]. Our attention was in any case focused on intermediate compartments more than on LV.

We observed that GFPChi was distributed essentially in the ER. The distribution was altered through auxin and ACh, both inducing the formation of small compartments labelled by GFPChi. AleuRFP pattern suffered no alteration. The small compartments labelled by GFPChi under auxin treatment were not an altered ER domain but colocalized with the PVC labelled by AleuRFP (Figure 4B). Also ACh induced the formation of discrete compartments. In this case small and large compartments were labelled. The small ones colocalized with AleuRFP-positive PVC (Figure 4C). The larger compartments may be defined small vacuoles by their size, larger than 1 μm. BFA treatment induced the formation of BFA bodies not very different from the “small vacuoles” induced by ACh, but colocalization of the two markers was never observed in the PVC.

We can speculate that both auxin and ACh, inhibiting a secondary step of endocytosis, prevent the formation of the intermediate compartment responsible for the correct sorting of GFPChi or, in the case of tomato cells, for its recovery to the ER. As a consequence, auxin and ACh cause the merging of GFPChi and AleuRFP sorting pathways and the colocalization of the two markers in the PVC. This effect may result in an indirect stimulation of the LV expansion and of cell expansion.

## 4. Experimental Section

### 4.1. Plant Material and Growth Conditions

Experiments were performed with 5-day-old tomato plants (*Solanum lycopersicon* cv. St. Pierre). Seeds of tomato were sown on half-strength Murashige and Skoog medium with 2 g/L Gelrite Gellan Gum (Sigma-Aldrich, Milan, Italy) in covered plastic sterile vessels (Duchefa Biochemie, Haarlem, The Netherlands), and then plants were grown in the dark for 5 days at 24 °C.

### 4.2. Hypocotyl Segment Treatment

Seedlings from 2.8 to 3.2 cm long were cut exactly below the apical hook in order to obtain one cm long segment. Measurement and cutting were conducted under a green safelight. Segments were incubated in Petri dishes containing 2.5 mM potassium phosphate buffer (pH 6.0) at 24 °C in the dark with gentle shaking as pre-treatment rinse in order to deplete endogenous auxin and other interfering putative signaling molecules [42,67]. After 2 h, the rinsing buffer was discarded and the segments were transferred into a new fresh buffer containing or not (control) different treatments (2,4-D (2,4-dichlorophenoxyacetic acid), sucrose, acetylcholine, atropine, tubocurarine) in different combinations. Sucrose and 2,4-D were purchased from Duchefa. Acetylcholine, atropine and tubocurarine were purchased from Sigma-Aldrich.

### 4.3. RNA Isolation and Reverse Transcription

Total RNA isolation was performed using RNAqueous kit (Applied Biosystems/Ambion, Monza, Italy), and then total RNA was DNase-treated using Turbo DNA-Free kit (Ambion) to remove possible DNA contamination. The quantity and quality of RNA samples were measured on a NanoDrop spectrophotometer (ND 1000, NanoDrop Technologies, Inc., Wilmington, DE, USA) at 260/280 nm. Reverse transcription was performed using ImProm-II Reverse Transcription System (Promega, Milan, Italy) and the cDNA concentration of each sample was normalised to 50 ng/μL prior to the qRT-PCR reactions.

### 4.4. Quantitative PCR

Quantitative-PCR (q-PCR) analysis was carried out using a TaqMan probe. The sequence used for the design of the probe for *LeEXPA2* gene was identified sequencing the fragment obtained using the primers described by Caderas *et al.* (2000) [41]. The probe consists of 248 bases with the target site for the probe located at position 186 of the sequence, while 18S rRNA was used as reference housekeeping gene because of its high stability [110] and lack of variation in the experimental treatments. Quantitative PCR was performed using the ABI PRISM(R) 7300 SequenceDetection System (Applied Biosystems, Foster City, CA, USA) in MicroAmp Optical 96-Well Reaction Plates. TaqMan Gene Expression Master Mix (Applied Biosystems) was used, containing AmpliTaq Gold DNA Polymerase. Data were evaluated in triplicate and the gene expression was calculated using the formula 2^−(ΔΔ^*^C^*^t)^.

### 4.5. FM4-64 Dye Staining and Transient Transformation of Hypocotyl Protoplasts

Protoplasts were obtained from hypocotyl segments grown as for the elongation assay. The manipulation, performed under normal white light, was performed as previously described for other plant material [55].

The FM4-64 staining was performed after 4 h of standard incubation at 26 °C. No transformation was performed in these cases. 100 μM FM4-64 (Molecular Probes, Leiden, The Netherlands) was used from a stock (1 mM) in 0.4 M mannitol. After 1 min the cells were washed with W5 (NaCl 154 mM; CaCls 125 mM; KCl 5 mM; Glucose 5 mM) and observed within 80 min. Images were produced as specified in the result description. The constructs GFPChi and AleuRFP, used for transient transformation, were built previously [100,102]. Protoplasts were examined with a confocal laser-microscope (LSM 710 Zeiss, ZEN software, GmbH, Jena, Germany). To detect FM4-64 fluorescence, the He-Ne laser was used to produce a 543-nm excitation and the emission was recorded with the 560–615 nm filter set. GFPChi was detected in the lambda range 505–530 nm, assigning the green colour, AleuRFP within 560–615 nm, assigning the red colour. Excitation wavelengths of 488 and 543 nm were used. The laser power was set to a minimum and appropriate controls were made to ensure there was no bleed-through from one channel to the other. Images were mounted using Adobe Photoshop 7.0 software (Mountain View, CA, USA).

## 5. Conclusions

Auxin and ACh effects on endomembranes seem to converge on the same traffic events but appear to be in any case distinct. The synergistic effect of auxin and ACh on *LeEXPA2* transcription and on hypocotyl elongation, associated with the observed diversified effect on endomembranes, supports the interpretation of two distinct signalling molecules capable of synergistic effects on elongation. A more accurate dissection of the molecular processes, supported by the increasing bibliography on auxin and brassinosteroids, has to be performed to fully explain the action of ACh in plants.
